# Financial-legal navigation reduces financial toxicity of pediatric, adolescent, and young adult cancers

**DOI:** 10.1093/jncics/pkae025

**Published:** 2024-03-29

**Authors:** Jean Edward, Kimberly D Northrip, Mary Kay Rayens, Andrea Welker, Rachel O’Farrell, Jennifer Knuf, Haafsah Fariduddin, Julia Costich, John D’Orazio

**Affiliations:** College of Nursing, University of Kentucky, Lexington, Kentucky, USA; Department of Pediatrics, College of Medicine, University of Kentucky, Lexington, Kentucky, USA; College of Nursing, University of Kentucky, Lexington, Kentucky, USA; Department of Pediatrics, College of Medicine, University of Kentucky, Lexington, Kentucky, USA; Danceblue Pediatric Hematology Oncology Clinic, Kentucky Children’s Hospital, University of Kentucky HealthCare, Lexington, Kentucky, USA; Danceblue Pediatric Hematology Oncology Clinic, Kentucky Children’s Hospital, University of Kentucky HealthCare, Lexington, Kentucky, USA; Markey STRONG, Markey Cancer Center, University of Kentucky HealthCare, Lexington, Kentucky, USA; Department of Health Management and Policy, College of Public Health, University of Kentucky, Lexington, Kentucky, USA; Department of Pediatrics, College of Medicine, University of Kentucky, Lexington, Kentucky, USA; Danceblue Pediatric Hematology Oncology Clinic, Kentucky Children’s Hospital, University of Kentucky HealthCare, Lexington, Kentucky, USA

## Abstract

**Background:**

Pediatric, adolescent, and young adult patients with cancer and their caregivers are at high risk of financial toxicity, and few evidence-based oncology financial and legal navigation programs exist to address it. We tested the feasibility, acceptability, and preliminary effectiveness of Financial and Insurance Navigation Assistance, a novel interdisciplinary financial and legal navigation intervention for pediatric, adolescent and young adult patients and their caregivers.

**Methods:**

We used a single-arm feasibility and acceptability trial design in a pediatric hematology and oncology clinic and collected preintervention and postintervention surveys to assess changes in financial toxicity (3 domains: psychological response/Comprehensive Score for Financial Toxicity [COST], material conditions, and coping behaviors); health-related quality of life (Patient-Reported Outcomes Measurement Information System Physical and Mental Health, Anxiety, Depression, and Parent Proxy scales); and perceived feasibility, acceptability, and appropriateness.

**Results:**

In total, 45 participants received financial navigation, 6 received legal navigation, and 10 received both. Among 15 adult patients, significant improvements in FACIT-COST (*P* = .041) and physical health (*P* = .036) were noted. Among 46 caregivers, significant improvements were noted for FACIT-COST (*P* < .001), the total financial toxicity score (*P* = .001), and the parent proxy global health score (*P* = .0037). We were able to secure roughly $335 323 in financial benefits for 48 participants. The intervention was rated highly for feasibility, acceptability, and appropriateness.

**Conclusions:**

Integrating financial and legal navigation through Financial and Insurance Navigation Assistance was feasible and acceptable and underscores the benefit of a multidisciplinary approach to addressing financial toxicity.

**ClinicalTrials.gov registration:**

NCT05876325

Key Points: *Question*: What are the feasibility and impact of a novel, interdisciplinary financial and legal navigation intervention on financial toxicity and health-related quality of life of pediatric, adolescent, and young adult oncology patients and their caregivers?

*Findings*: In this single-arm feasibility and acceptability trial, 15 adult patients and 43 caregivers experienced significant improvements in financial toxicity and health-related quality of life after participating in the intervention.

*Meaning*: Integrating a comprehensive oncology financial and legal navigation program into oncology care can improve health and financial outcomes for patients and their caregivers.

Pediatric, adolescent, and young adult patients with cancer and their caregivers are at high risk of experiencing financial toxicity—that is, the financial burden and stress related to cancer costs, especially in low-income and rural regions (1-3). The financial burden of cancer care is exacerbated by financial and sociolegal barriers such as unstable or unsafe housing, lack of adequate health insurance, or loss of job and income (4-7). Enhancing patient–health-care professional cost-of-care conversations can be an effective strategy to identify and address these health-harming sociolegal needs (8,9). Lack of knowledge, skills, and time often prevent health-care teams from fully engaging in cost-of-care conversations, resulting in a lack of financial and legal navigation to comprehensively address patients’ sociolegal needs (10). Team-based approaches that integrate the expertise of clinical, social work, financial, and legal experts at the point of care can enhance cost-of-care conversations and improve outcomes for patients.

One such approach is a medical-legal partnership. These partnerships are evidence-based interventions that integrate lawyers into the health-care setting to address sociolegal needs (11,12). Medical-legal partnerships serve as a model both to facilitate and to manage outcomes of cost-of-care conversations. Lawyers are uniquely positioned to address families’ financial and sociolegal barriers by advising families on how to gain access to available support or by providing civil legal representation to directly address those barriers when a legal remedy exists. To address the full spectrum of sociolegal needs, however, interdisciplinary team-based approaches that include clinicians, social workers, and financial navigators as well as lawyers are essential (13-15). The impact of medical-legal partnerships on improving sociolegal needs of underserved populations is well documented (11), but evidence is limited on their impact on cancer-related financial toxicity. To address these gaps, we tested the feasibility, acceptability, and preliminary effectiveness of Financial and Insurance Navigation Assistance (FINassist), a novel interdisciplinary financial and legal navigation intervention that uses medical-legal partnerships to enhance cost-of-care conversations and reduce financial toxicity for pediatric oncology patients and their caregivers.

## Methods

### Study design, setting, and participants

We conducted a single-arm feasibility and acceptability trial in the University of Kentucky Division of Pediatric Oncology and Hematology’s DanceBlue Clinic (ClinicalTrials.gov identifier NCT05876325). The clinic is affiliated with Markey Cancer Center, a National Cancer Institute–Designated Cancer Center, which serves approximately 300 pediatric, adolescent, and young adult oncology and hematology patients per month and roughly 80 new pediatric cancer cases per year. The clinic also serves a large rural population. Adult (aged 18 years and older) patients and caregivers of patients younger than 18 years of age with any pediatric, adolescent, or young adult cancer or hematologic disorder diagnosis were recruited from the clinic. Participants provided written informed consent before collection of survey and electronic health record data. The study was approved by the University of Kentucky’s Institutional Review Board (No. 57238).

### The *FINassist* intervention

Development and evaluation of the FINassist intervention were guided by the Consolidated Framework for Implementation Research (16); findings from phase 1 of this study (17); and feedback from our clinical team, patient and study advisory boards, and consultants. FINassist is built on a platform of interdisciplinary team–based science and includes the equal participation of clinicians, social workers, financial navigators, and lawyers in enhancing cost-of-care conversations to address financial toxicity. Our model is based on the premise that it takes an interdisciplinary team to facilitate effective cost-of-care conversations that address complex sociolegal issues affecting the health of patients with pediatric, adolescent, and young adult cancers.

**Figure 1.**
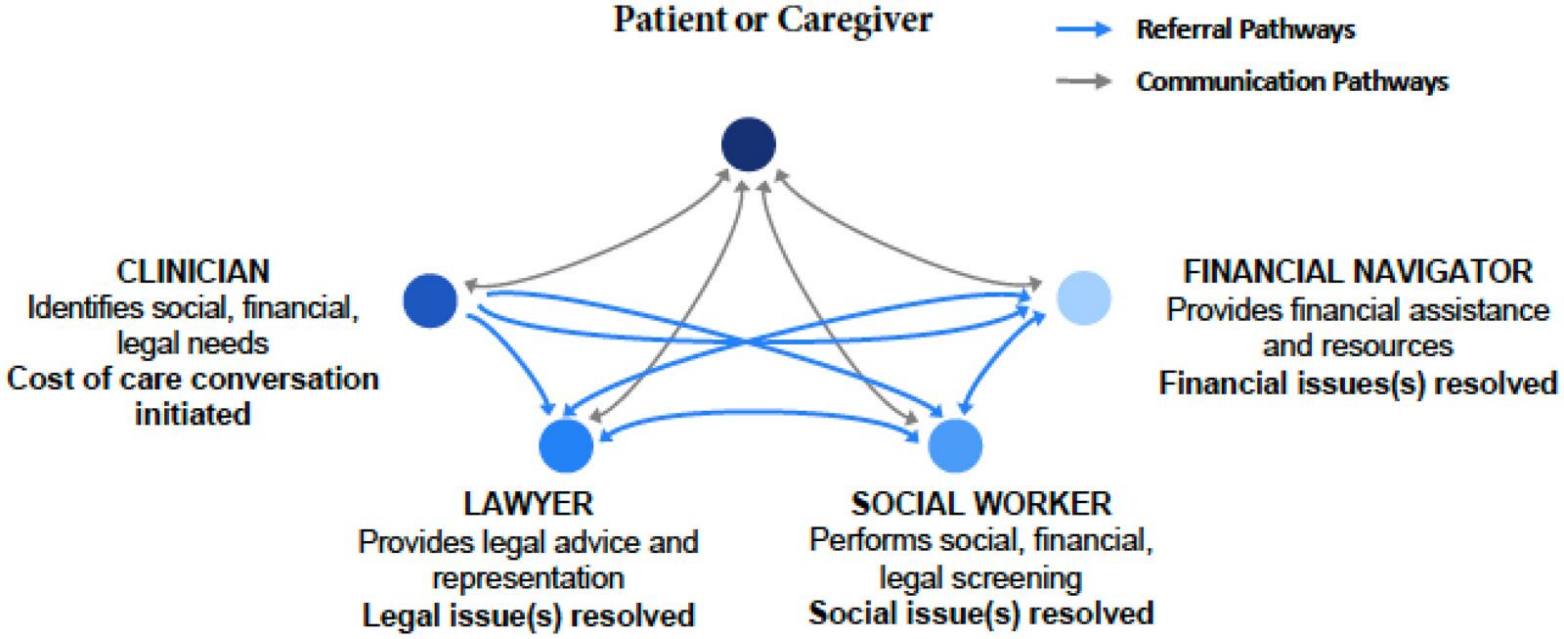
This model depicts the workflow and communication pathways used in the Financial and Insurance Navigation Assistance intervention.

Figure 1 depicts the workflow of communication, referrals, and navigation used in FINassist. The timing, delivery, and messaging of the intervention were designed to ensure that patients and caregivers had multiple opportunities to engage in cost-of-care conversations as they move through the cancer care continuum. All patients and caregivers who visited the clinic between March 2021 and May 2022 received information about the study through informational brochures and flyers. Members of the health-care team were trained in engaging in cost-of-care conversations, identifying needs, and recruiting before implementation of FINassist. Clinicians used a brief script to initiate cost-of-care conversations and introduced FINassist to all patients and caregivers. Social workers followed up with sociolegal needs screening. If families indicated having any sociolegal needs, social workers briefly introduced resources available through FINassist and obtained informed consent before collecting presurveys and referring to the appropriate FINassist team member. The financial navigator helped resolve billing issues related to insurance or Medicaid claims and connected participants to financial resources such as grants, charity care, and financial assistance programs. Lawyers were contracted through an medical-legal partnership from a local legal aid organization to help resolve legal issues related to health and social benefits, housing, employment, education, immigration, and family law.

### Measures

#### Demographic and clinical data

Adult patients and caregivers completed a sociolegal needs screener and demographic survey that provided data on variables, including sex, age, race and ethnicity, relationship to patient (if a caregiver), education, income, perceived income sufficiency, occupational status, insurance type, and most frequent financial or legal concerns. Additional patient demographic and clinical variables were extracted from the electronic health record, including age at diagnosis, sex, race and ethnicity, diagnosis, treatments, and medications.

#### Patient-reported and caregiver-reported financial toxicity outcomes

Financial toxicity was measured in 3 domains (18): psychological response, material conditions, and coping behaviors. Higher scores in these domains reflect higher financial toxicity, except for scores for psychological response, where lower scores indicated a higher financial toxicity. Psychological response was measured using the COST: A FACIT Measure of Financial Toxicity (FACIT-COST) (19), an 11-item scale, with each item scored on an ordinal scale from 0 (“not at all”) to 4 (“very much”). Negative items are reverse-coded before creating the total FACIT-COST score. Internal consistency has been reported as α = .92 (19). Material conditions were measured based on 8 items, including 6 from the Medical Expenditure Panel Survey–Experiences with Cancer Survivorship Supplement (20) and 6 from the demographic survey, on direct costs of cancer care, including indicators for borrowing money, going into debt, and filing for bankruptcy. Items were summed to create a total score. Coping behaviors were measured using a series of 16 yes/no supplement (20) items related to foregoing care due to financial circumstances. A total score was obtained by determining the number of “yes” responses so that higher scores indicate greater financial toxicity. Typical items include indicators for whether they have gone without care because they could not afford it, because the doctor did not accept their insurance, or because they could not get time off from work. Item selection and scoring for material conditions and coping behaviors were based on a previous study (21).

A total financial toxicity score was created from the 3 domains of psychological response, material conditions, and coping behaviors (21). To give the psychological response domain the same polarity as the others, the FACIT-COST was reverse-scored in the calculation. For each domain, observed total scores were divided by the maximum possible total to create a percentile score for each instrument; these percentiles were summed to form the total financial toxicity measure. The potential range was 0 to 3, with higher scores indicating greater financial toxicity.

Health-related quality of life (QOL) was measured using the Patient-Reported Outcomes Measurement Information System (PROMIS) physical health and mental health subscales (from the 10-item PROMIS Scale, version 1.2–Global Health) (22), the 4-item PROMIS Anxiety Short Form (23), and the 6-item PROMIS Depression Short Form (24). The PROMIS Global Health Scale 7 + 2–Parent Proxy (25) was used to measure child QOL (when applicable), with summary scores for proxy measures of global health, pain interference, and fatigue. PROMIS scoring was conducted in HealthMeasures; standardized *t* scores were used for analysis.

#### Implementation outcomes measures

Feasibility was defined as 60% or higher enrolled participant retention and resolution of financial or legal issue (based on existing oncology financial navigation studies) (21). In addition we measured perceived implementation outcomes of feasibility, acceptability and appropriateness (26). Each of these 3 measures was formed by summing 4 items, and the response set per item ranged from 0 (completely disagree) to 4 (completely agree). The total scores for the subscales had potential ranges of 0 to 16, with higher scores indicating a more positive perception of each FINassist attribute.

### Data analysis

Patient and caregiver demographic and clinical variables were summarized using descriptive statistics, including means (SD) or frequency distributions. The key outcome variables included the financial toxicity total score and domain totals (for psychological response, material conditions, and coping behaviors); the PROMIS measures of physical and mental health, anxiety, and depression; and the PROMIS parent proxy measures of global health, pain interference, and fatigue. These variables were summarized at baseline using means and SDs in addition to the estimate for the percentage of the maximum possible score the corresponding mean represented. Finally, paired *t* tests were used to evaluate changes between preintervention and postintervention outcomes for the adult patients and the adult caregivers of pediatric patients separately. An ɑ = 2.05 was used for these comparisons between before and after scores.

## Results

### Demographic and clinical data

**Figure 2.**
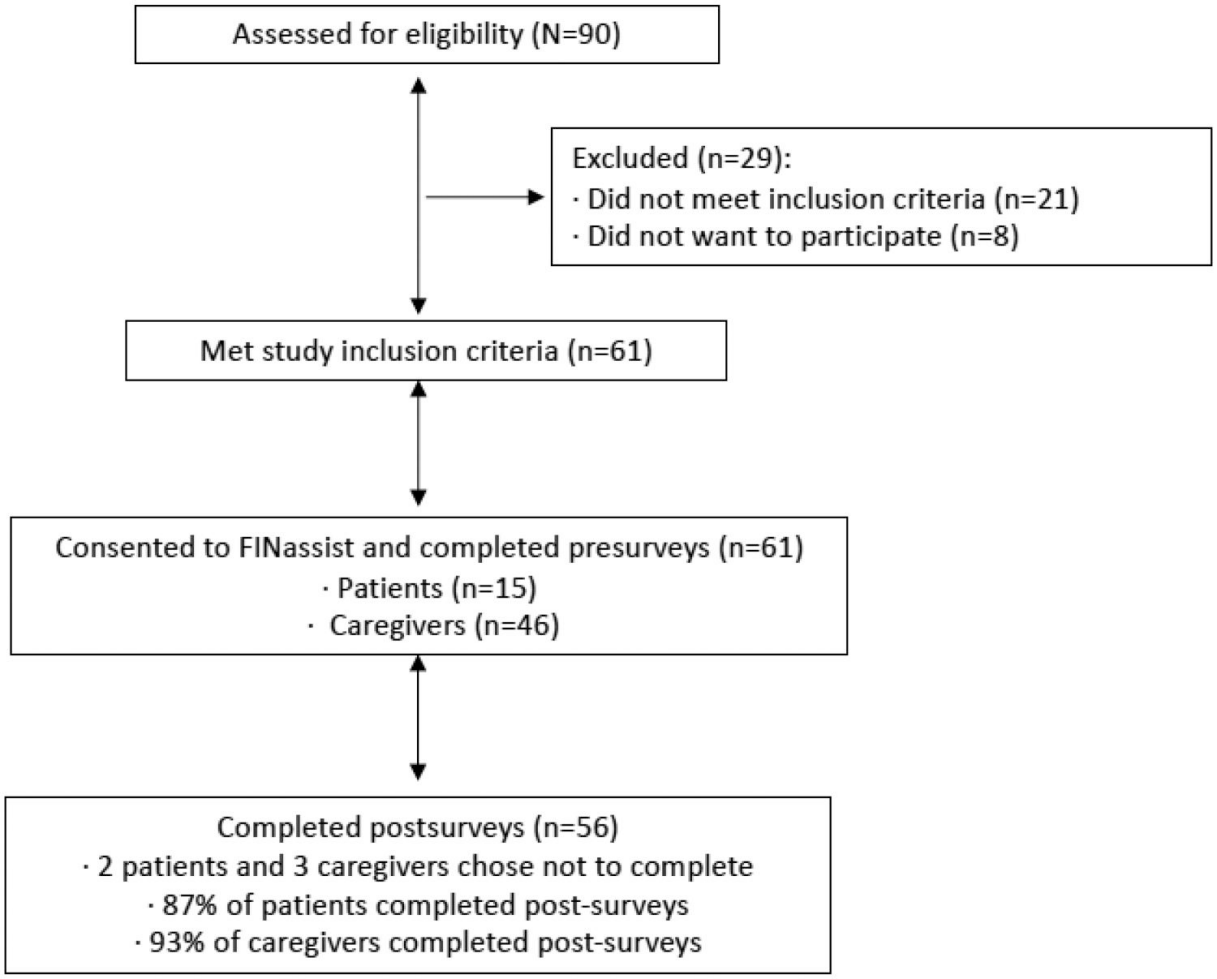
This CONSORT flow diagram shows the progress through the phases of this clinical trial. FINassist = Financial and Insurance Navigation Assistance.

Fifteen adult patients with a diagnosed pediatric, adolescent, or young adult cancer or hematologic disorder and 46 caregivers enrolled in FINassist (see Figure 2). As shown in Table 1, only one-third of adult patients were female (33%) compared with more than three-quarters of caregivers (80%). Average age for adult patients and caregivers was 33.5 and 39.3 years, respectively. Although both the adult patient and caregiver groups were predominantly White and non-Hispanic, 40% of the adult patients identified as Black/non-Hispanic or Hispanic. Most of the caregivers were parents (96%). The majority (71%) of caregivers had some postsecondary education compared with 33% of adult patients.

**Table 1.** Descriptive summary of baseline demographic variables for adult patients and caregivers of pediatric patients (N* *=* *61)

Variable	Adult patients	Caregivers
	(n* *=* *15)	(n* *=* *46)
Female sex, No. (%)	5 (33.3)	37 (80.4)
Age, mean (SD) [range], y	33.5 (16.2) [19-69]	39.3 (10.2) [22-60]
Race and ethnicity, No. (%)		
White, non-Hispanic	9 (60.0)	39 (84.8)
Black, non-Hispanic	4 (26.7)	4 (8.7)
Hispanic	2 (13.3)	3 (6.5)
Relationship to patient, No. (%)		
Self	15 (100.0)	0 (0.0)
Parent	0 (0.0)	44 (95.7)
Guardian	0 (0.0)	2 (4.3)
Education, No. (%)		
High school diploma or less	10 (66.7)	13 (28.9)
At least some postsecondary education	5 (33.3)	32 (71.1)
At or below federal poverty level, No. (%)	6 (46.2)	12 (30.0)
How does your present income feel? No. (%)		
Living comfortably	4 (26.7)	6 (13.0)
Getting by	5 (33.3)	31 (67.5)
Finding it difficult	5 (33.3)	6 (13.0)
Finding it very difficult	1 (6.7)	3 (6.5)
Occupational status, No. (%)		
Employed	5 (33.3)	31 (68.9)
Unemployed, disabled, or medical leave	9 (60.0)	9 (20.0)
Homemaker	0 (0.0)	5 (11.1)
Retired	1 (6.7)	0 (0.0)
Insurance type, No. (%)		
None (uninsured)	3 (20.0)	0 (0.0)
Plan purchased through employer or union	3 (20.0)	25 (54.4)
Plan you bought on your own	0 (0.0)	1 (2.1)
Medicaid, Children’s Health Insurance Program, or similar	9 (60.0)	20 (43.5)
Most frequent financial concerns, No. (%)		
Paying for health-care bills	12 (80.0)	31 (67.4)
Current financial situation, given health-care needs	12 (80.0)	26 (56.5)
Health insurance	11 (73.3)	22 (47.8)
Accessing disability benefits	5 (33.3)	14 (30.4)
Lack of reliable transportation	6 (42.9)	3 (4.5)
Most frequent legal concerns, No. (%)		
Employment	10 (66.7)	24 (52.2)
Housing	4 (26.7)	12 (26.1)
Education	3 (20.0)	12 (26.1)
Family	1 (6.7)	10 (21.7)
Immigration	1 (6.7)	0 (0.0)

Nearly half (46%) of the adult patients were at or below the federal poverty level compared with 30% of caregivers. Among adult patients, 40% found it difficult to very difficult to make ends meet financially compared with 20% of caregivers. Adult patients were more likely (60%) to report that they were unemployed, disabled, or on medical leave compared with caregivers (20%). Most adult patients (60%) were covered by Medicaid, Children’s Health Insurance Program, or similar, while caregivers were most frequently (54%) covered by a plan purchased through an employer. Both adult patients and caregivers shared many of the same financial concerns, with most frequent concerns related to paying for health care.

The demographic summary of the 15 adult patients and 47 pediatric patients of caregivers enrolled in the study, taken from the electronic health record, demonstrated that the average (SD) age at diagnosis was 6.4 (5.9) years (range = 0-18). Slightly more than half (55%) identified as male, and most (78%) identified as White and non-Hispanic. Five (8%) had a cancer diagnosis, 17 (27%) had a hematologic disorder, and 40 (65%) had cancer plus hematologic sequela (Table 2). Nearly half (n = 29 [47%]) had received at least 1 blood transfusion, and more than two-thirds (n = 43 [69%]) had received 6 or more chemotherapy treatments.

**Table 2.** Descriptive summary of diagnoses, treatments, and procedures recorded in the pediatric and adult patients’ electronic health records (N* *=* *62)

Variable	No. (%)^a^	Average No.^b^ (range)
Diagnosis		
Cancer alone	5 (8.1)	—
Blood-related disease alone	17 (27.4)	—
Both cancer and blood-related disease	40 (64.5)	—
Treatment received		
≥1 blood transfusions	29 (46.8)	9.1 (1-70)
≥6 chemotherapy treatments	43 (69.4)	75.3 (6-328)
Procedure received		
≥1 bone marrow biopsies or aspirations	27 (43.5)	2.3 (1-6)
≥1 electrocardiograms	26 (41.9)	3.2 (1-13)
≥1 lumbar punctures	10 (16.1)	1.2 (1-2)
≥1 venous access, drug delivery, irrigations	18 (29.0)	3.6 (1-17)

a The percentages for treatments and procedures do not sum to 100 because a given patient could have received more than 1 type of each; the percentage reported for each category is relative to the total number of patients (N = 62). One caregiver had 2 children.

b Average number received per person among those who received any and range in per-person totals.

### FINassist delivery and processes

Forty-five participants received financial navigation only, 6 received legal navigation only, and 10 received both. The most common financial concerns were related to paying for medical bills, financial circumstances, and health insurance (Table 1). Common legal concerns were related to employment, housing, and education. One-on-one interactions with the financial navigator and lawyers ranged from 5 to 20 sessions, with most requiring ongoing assistance. We observed a 91.8% participant retention rate, with 100% resolution of financial issues and 68.7% (n = 11) resolution of legal issues (3 client cases were pending upon study closure, 1 client did not follow through with services, and 2 clients lived outside the legal aid service area and were referred to the appropriate organization). The financial navigator secured roughly $335 323 in financial benefits for 48 participants by addressing Medicaid or insurance issues ($145 329) and billing errors ($97 837) as well as through financial assistance programs and grants ($83 807) and food stamps ($8350).

### Patient-reported and caregiver-reported outcomes

As shown in Table 3, adult patients and caregivers had mean total financial toxicity scores that were 29% to 35% of the maximum toxicity of 3. Both groups reported higher financial toxicity in the psychological response domain (FACIT-COST), with adult patients and caregivers having a mean score that was 40% to 48% of the of the maximum possible score, respectively. Physical and mental QOL were somewhat worse for adult patients (59% of maximum physical QOL compared with 71% for caregivers and 60% of maximum mental QOL compared with 67% for caregivers), but both groups reported similar anxiety and depression scores. The parent-proxy global health scores were a little over half of the maximum possible score (58%), on average. The pain interference and fatigue means indicated the presence of significant symptoms at between 79% and 82% of the total possible scores for each measure.

**Table 3.** Mean (SD) for baseline outcomes among adult patients and caregivers (N* *=* *61)

	Adult patients (n = 15)	Caregivers (n = 46)
Variable	Mean (SD); range [maximum possible] <mean as % of maximum>	Mean (SD); range [maximum possible] <mean as % of maximum>
Total financial toxicity score**^a^**	1.06 (0.55); 0.22-2.36 [3]	0.88 (0.30); 0.30-1.50 [3]
	<35.3>	<29.3>
Financial toxicity: psychological response (FACIT-COST)	17.50 (9.07); 4-34 [44]	21.11 (6.45); 4-35 [44]
	<39.8>	<48.0>
Financial toxicity: material conditions	2.37 (1.62); 0-4.67 [7]	2.43 (1.34); 0.33-5.33 [7]
	<33.9>	<34.7>
Financial toxicity: coping behaviors	0.71 (1.90); 0-7 [8]	0.087 (0.35); 0-2 [8]
	<8.9>	<1.1>
PROMIS Physical Health	40.08 (4.52); 31.3-46.3 [67.7]	47.76 (8.51); 26.6-67.7 [67.7]
	<59.2>	<70.5>
PROMIS Mental Health	40.57 (8.02); 25.0-50.9 [67.6]	45.12 (8.39); 25.0-67.6 [67.6]
	<60.0>	<66.7>
PROMIS Anxiety	55.69 (9.10); 40.3-69.5 [81.4]	58.14 (7.84); 40.3-78.0 [81.4]
	<68.4>	<71.4>
PROMIS Depression	54.42 (10.27); 38.4-68.0 [79.6]	53.38 (8.79); 38.4-77.3 [79.6]
	<68.4>	<67.1>
PROMIS Parent Proxy Global Health	—	38.45 (10.01); 18.0-58.0 [66.1]
	—	<58.2>
PROMIS Parent Proxy Pain Interference	—	54.25 (7.74); 43.3-68.8 [68.8]
	—	<78.9>
PROMIS Parent Proxy Fatigue	—	55.93 (8.16); 40.1-68.1 [68.1]
	—	<82.1>

a Financial toxicity total score includes average of reverse-coded FACIT-COST, material condition, and coping behavior scores. FACIT = Functional Assessment of Chronic Illness Therapy; FACIT-COST = COST: A FACIT Measure of Financial Toxicity; PROMIS = Patient-Reported Outcomes Measurement Information System.

Among adult patients (n* *=* *15), there were significant improvements from before to after the intervention for psychological response (FACIT-COST*; P* = .041) and physical health (*P* = .036) (Table 4). Among caregivers (n* *=* *46), the improvement from before to after the intervention was significant for total financial toxicity (*P* < .001) and psychological response (*P* = .001). The parent proxy global health score also increased significantly (*P* = .0037) from baseline to the after the intervention.

**Table 4.** Paired *t* tests of study outcomes for the adult patient and caregiver samples

Variable	Adult patients (n* *=* *15)		Caregivers (n* *=* *46)	
	**Mean (SD)** ^a^	Paired *t* (*P*)	Mean (SD)	Paired *t* (*P*)
Total financial toxicity score**^b^**	0.17 (0.34)	1.68 (.12)	0.14 (0.26)	3.56 (.001)
Financial toxicity: psychological response (FACIT-COST)	‒4.92 (7.37)	2.31 (.041)	‒5.34 (6.47)	5.48 (<.001)
Financial toxicity: material conditions	0.42 (1.32)	1.10 (.30)	0.26 (1.28)	1.28 (.21)
Financial toxicity: coping behaviors	0.00 (1.29)	<0.10 (>.99)	‒0.045 (0.37)	0.81 (.42)
PROMIS Physical	‒2.95 (4.51)	2.36 (.036)	‒1.58 (6.70)	1.55 (.13)
PROMIS Mental	‒2.08 (4.10)	1.83 (.092)	‒0.91 (8.43)	0.71 (.48)
PROMIS Anxiety	‒0.35 (7.76)	0.16 (.87)	2.38 (8.40)	1.86 (.071)
PROMIS Depression	0.077 (10.19)	<0.10 (.98)	1.79 (8.52)	1.38 (.18)
PROMIS Parent Proxy Global Health	—	—	‒3.88 (8.37)	3.07 (.0037)
PROMIS Parent Proxy Pain Interference	—	—	1.91 (9.43)	1.35 (.19)
PROMIS Parent Proxy Fatigue	—	—	2.07 (8.04)	1.69 (.099)

a Each difference is defined as the postintervention score subtracted from the preintervention score, and average change scores are also shown in the table. FACIT = Functional Assessment of Chronic Illness Therapy; FACIT-COST = COST: A FACIT Measure of Financial Toxicity; PROMIS = Patient-Reported Outcomes Measurement Information System.

b Financial toxicity total score includes average of reverse-coded FACIT-COST (so all measures have the same polarity), material condition, and coping behavior scores.

### FINassist implementation outcomes

Participants, including adult patients and caregivers of pediatric patients, rated the FINassist program favorably. For the 56 participants who completed the postintervention survey, the average (SD) overall intervention evaluation score was 53.8 (11.6) (range = 6-60), with high feasibility at 14.0 (2.4) (range = 4-16), acceptability at 13.8 (2.8) (range = 2-16), and appropriateness at 14.0 (2.5) (range = 4-16) ratings.

## Discussion

To our knowledge, this study is the first to evaluate the feasibility, acceptability, and preliminary effectiveness of a combined financial and medical-legal partnership–based legal navigation intervention in a pediatric, adolescent, and young adult oncology and hematology setting. After participation in FINassist, we found improvements in the psychological response domain of financial toxicity (and the total financial toxicity score for caregivers), which were relatively high at baseline for adult patients and caregivers enrolled in the study. The intervention was also associated with improved physical health (adult patients) and parent-proxy Global Health scores for pediatric patients.

High baseline financial toxicity measurements for adult patients and caregivers enrolled in our study are consistent with prior research (27,28). Previous studies have also highlighted the benefit of financial (21,29) or legal (20,31) navigation in mitigating the economic burden associated with adult cancer care. Our study addresses gaps in the literature on financial toxicity in the pediatric, adolescent, and young adult oncology patient and caregiver population (5) by providing a better understanding of the financial and legal challenges that lie within this population. Additionally, this was 1 of the first studies to capture the impact of financial and legal navigation on the FACIT-COST in the pediatric, adolescent, and young adult oncology population.

Current medical-legal partnership literature lacks evidence of interventional impact on patient-reported financial toxicity outcomes in the pediatric oncology patient and caregiver population (28,30). Our study remedies this gap by quantifying the benefit of the FINassist intervention in this population. Although our evaluation of medical-legal partnership in a pediatric oncology and hematology setting is novel, our results align with existing research that medical-legal partnerships have had positive impacts on psychosocial distress, health-related QOL, and other patient outcomes in other health-care settings (9,10,30,31). Battaglia et al. (31) implemented a legal navigation intervention in an adult oncology setting and found that 75% of patients faced at least 1 sociolegal barrier to care, highlighting the importance of incorporating a legal perspective when establishing interventions in this setting. In providing essential support, medical-legal partnerships alleviate stressors and empower patients and caregivers to focus on their treatment and overall well-being. Additional research is needed, however, to help support the efficacy of medical-legal partnerships, especially as they pertains to influencing financial toxicity.

FINassist added financial navigation services to the medical-legal partnership model. To our knowledge, only 2 other studies have examined combined financial and legal interventions (29,32). Both studies reported enhanced effectiveness when they implemented frequent participant-navigator interactions and were able to address a broad spectrum of financial and legal needs (29,32). A common denominator among all these studies was the high levels of financial burden and anxiety that participants initially reported. Despite including some type of legal aid, few financial navigation studies have established and evaluated a formal medical-legal partnership as we did in the current study. FINassist’s integration of financial and legal navigation into health-care services underscores the benefit of a multidisciplinary approach to cost-of-care conversations. The medical-legal partnership identified and addressed legal issues that otherwise would not have been considered, and the financial navigator provided financial benefits by resolving insurance barriers and facilitating access to financial support that would not be provided in a typical health-care setting.

In addition to quantifying benefit, this study demonstrated the feasibility of a combined financial navigation and medical-legal partnership model in a pediatric, adolescent, and young adult hematology-oncology setting. The study succeeded in retaining participants and was evaluated favorably by patients and caregivers for feasibility, acceptability, and appropriateness. A diverse sample of adult patients and caregivers of pediatric patients highlights the intervention’s range of impact. These insights suggest the possibility of scaling this intervention for a broader population.

Having demonstrated the feasibility, acceptability, and preliminary outcomes of the FINassist intervention, our next step would be to test it in a randomized controlled trial to further evaluate its effectiveness. Alternative models for delivering oncology legal navigation should be explored, as well, including the possibility of employing in-house lawyers in the same manner as financial navigators as opposed to collaborating with local legal aid organizations.

Establishing an medical-legal partnership requires close collaborations between legal entities and health-care teams that often necessitates lawyers co-locating within clinic spaces (30,31). As a result of the pandemic, our medical-legal partnership lawyers could not be onsite to work in tandem with health-care teams. This situation negatively affected referrals and communication during the study and limited the study. A second limitation was sample size: The study was not powered to look at the adult patient group alone. The small sample size and single postassessment vehicle limit the generalizability and assessment of the long-term impact of the intervention.

To our knowledge, this study is the first to assess the preliminary effectiveness of an medical-legal partnership–incorporated financial and legal intervention in a pediatric oncology-hematology setting. The FINassist intervention was associated with reduced financial toxicity for both adult patients and caregivers enrolled in the study and with improved physical health assessments. The intervention demonstrated high feasibility, acceptability, and appropriateness. Therefore, further study of incorporating medical-legal partnerships into the oncology setting is warranted because medical-legal partnerships hold great potential for enhancing patient care and outcomes. At the core of a comprehensive care approach is collaboration among various services and teams, enabling a multifaceted response to patient and caregiver needs. This collaboration is necessary to improve QOL, and the FINassist intervention uses this synergy to its advantage.

## Data Availability

Research data are not shared due to existing consent and Institutional Review Board constraints.
